# A precise performance-based reimbursement model for the multi-centre NAPKON cohorts – development and evaluation

**DOI:** 10.1038/s41598-024-63945-5

**Published:** 2024-06-13

**Authors:** Katharina S. Appel, Chin Huang Lee, Susana M. Nunes de Miranda, Daniel Maier, Jens-Peter Reese, Gabriele Anton, Thomas Bahmer, Sabrina Ballhausen, Beate Balzuweit, Carla Bellinghausen, Arne Blumentritt, Markus Brechtel, Irina Chaplinskaya-Sobol, Johanna Erber, Karin Fiedler, Ramsia Geisler, Ralf Heyder, Thomas Illig, Mirjam Kohls, Jenny Kollek, Lilian Krist, Roberto Lorbeer, Olga Miljukov, Lazar Mitrov, Carolin Nürnberger, Christian Pape, Christina Pley, Christian Schäfer, Jens Schaller, Mario Schattschneider, Margarete Scherer, Nick Schulze, Dana Stahl, Hans Christian Stubbe, Thalea Tamminga, Johannes Josef Tebbe, Maria J. G. T. Vehreschild, Silke Wiedmann, Jörg Janne Vehreschild

**Affiliations:** 1 Goethe University Frankfurt, University Hospital, Center for Internal Medicine, Medical Department 2 (Hematology/Oncology and Infectious Diseases), Theodor-Stern-Kai 7, 60596 Frankfurt, Germany; 2 University of Cologne, Faculty of Medicine and University Hospital of Cologne, Department I for Internal Medicine, Cologne, Germany; 3 German Cancer Consortium (DKTK), Partner Site Frankfurt/Mainz and German Cancer Research Center (DKFZ), Heidelberg, Germany; 4 University of Würzburg, Institute for Clinical Epidemiology and Biometry, Würzburg, Germany; 5 University Hospital Würzburg, Institute for medical Data Science, Würzburg, Würzburg, Germany; 6 Medical School OWL, Bielefeld University, Bielefeld, Germany; 7 Internal Medicine Department I, Pneumology Section, University Hospital Schleswig-Holstein Campus Kiel, Kiel, Germany; 8 German Center for Lung Research (DZL), Airway Research Center North (ARCN), Grosshansdorf, Germany; 9 Charité – Universitätsmedizin Berlin, Corporate Member of Freie Universität Berlin and Humboldt-Universität zu Berlin, Department of Infectious Diseases, Respiratory Medicine and Critical Care, Berlin, Germany; 10 Department I of Internal Medicine, Goethe University Frankfurt, University Hospital Frankfurt, Frankfurt, Germany; 11 Independent Trusted Third Party of the University Medicine Greifswald, Ellernholzstraße 1-2, 17475 Greifswald, Germany; 12 Department of Medical Informatics at the University Medical Center Göttingen, Göttingen, Germany; 13 TUM School of Medicine and Health, Department of Clinical Medicine, Clinical Department for Internal Medicine II, Technical University of Munich, University Medical Center, Munich, Germany; 14 Charité – Universitätsmedizin Berlin, corporate member of Freie Universität Berlin and Humboldt-Universität zu Berlin, NUM Coordination Office, Charitéplatz 1, 10117 Berlin, Germany; 15 Hannover Medical School, Hannover Unified Biobank, Hannover, Germany; 16 Charité - Universitätsmedizin Berlin, Corporate Member of Freie Universität Berlin and Humboldt-Universität zu Berlin, Department of Infectious Diseases, Respiratory Medicine and Critical Care, Clinical Trial Unit Berlin, Berlin, Germany; 17 Institute of Social Medicine, Epidemiology and Health Economics, Charité – Universitätsmedizin Berlin, corporate member of Freie Universität Berlin and Humboldt-Universität zu Berlin, Berlin, Germany; 18 Deutsches Herzzentrum der Charité, Institute of Computer-Assisted Cardiovascular Medicine, Berlin, Germany; 19 Charité – Universitätsmedizin Berlin, corporate member of Freie Universität Berlin and Humboldt Universität zu Berlin, Berlin, Germany; 20 Department of Radiology, Ludwig-Maximilians-University Hospital, Munich, Germany; 21 University of Cologne, Faculty of Medicine and University Hospital of Cologne, Department I for Internal Medicine, Center for Integrated Oncology Aachen Bonn Cologne Duesseldorf, Cologne, Germany; 22 Charité – Universitätsmedizin Berlin, corporate member of Freie Universität Berlin and Humboldt-Universität zu Berlin, Clinical Trial Office, Berlin, Germany; 23 Institute of Clinical Chemistry and Laboratory Medicine, University Medicine Greifswald, Greifswald, Germany; 24 Department of Medicine II, University Hospital, LMU Munich, Munich, Germany; 25 German Center for Infection Research (DZIF), Partner-Site Munich, Munich, Germany; 26 Hospital Lippe, Department of Gastroenterology and Infectious Diseases, Lippe, Germany; 27 Bielefeld University, Medical School OWL, Bielefeld, Germany; 28 Goethe University Frankfurt, University Hospital Frankfurt, Department II of Infectious Diseases, Frankfurt, Germany; 29 German Center for Infection Research (DZIF), Partner-Site Cologne-Bonn, Cologne, Germany

**Keywords:** Performance-based reimbursement, Funding allocation, Clinical studies, COVID-19, Research infrastructure, Germany, Clinical trial design, Infectious diseases

## Abstract

Fair allocation of funding in multi-centre clinical studies is challenging. Models commonly used in Germany - the case fees (“fixed-rate model”, FRM) and up-front staffing and consumables (“up-front allocation model”, UFAM) lack transparency and fail to suitably accommodate variations in centre performance. We developed a performance-based reimbursement model (PBRM) with automated calculation of conducted activities and applied it to the cohorts of the National Pandemic Cohort Network (NAPKON) within the Network of University Medicine (NUM). The study protocol activities, which were derived from data management systems, underwent validation through standardized quality checks by multiple stakeholders. The PBRM output (first funding period) was compared among centres and cohorts, and the cost-efficiency of the models was evaluated. Cases per centre varied from one to 164. The mean case reimbursement differed among the cohorts (1173.21€ [95% CI 645.68–1700.73] to 3863.43€ [95% CI 1468.89–6257.96]) and centres and mostly fell short of the expected amount. Model comparisons revealed higher cost-efficiency of the PBRM compared to FRM and UFAM, especially for low recruitment outliers. In conclusion, we have developed a reimbursement model that is transparent, accurate, and flexible. In multi-centre collaborations where heterogeneity between centres is expected, a PBRM could be used as a model to address performance discrepancies.

Trial registration: https://clinicaltrials.gov/ct2/show/NCT04768998; https://clinicaltrials.gov/ct2/show/NCT04747366; https://clinicaltrials.gov/ct2/show/NCT04679584.

## Introduction

Collaborative research is becoming increasingly important in multi-centre clinical trials. Sharing expertise and resources enhance scientific opportunities^1,2^. Along with increasing complexity and scope of collaborative projects, the need for efficient project management and infrastructure rises^1,3^. Despite the obvious need for improvement, only few studies evaluate requirements and challenges of collaborative research^2^. One key aspect is transparent and fair allocation of funding to the involved partners^4^.

In Germany, collaborative clinical studies (excluding pharmaceutical trials) are usually reimbursed by up-front payments for staff and consumables (up-front allocation model, UFAM) relying on a predefined number of expected cases, or post-hoc case fees (fixed-rate model, FRM) for each recruited participant. Both models typically rely on experience-based assumptions about the workload and material expenses connected with protocol-defined study activities; adjustment mechanisms to meet actual requirements over the course of the project are often lacking. The models assume that the average resource consumption for conducting the study is similar across all participating centres and that all centres recruit enough subjects to compensate given variations. However, these assumptions have significant limitations regarding application, flexibility, and cost-efficiency (i.e. the concept of economic viability of activities). For instance, due to variability in case severity^5–7^, observation duration, drop-out rates, participant compliance, or protocol adherence, the required resources to carry out a study can differ significantly between participants and centres. This may lead to substantial funding deviation for either the receiving or providing parties under the two commonly used models.

In 2020, we were tasked with allocating the budget for the German National Pandemic Cohort Network (Nationales Pandemie Kohorten Netz, NAPKON)^8^ among all 36 university hospitals as well as other non-academic hospitals and private practices. To allow fair reimbursement of study centre activities while maintaining accountability for principal investigators and the funding agency in the setting of an emerging new disease, we designed a method for the allocation of financial resources to participating centres according to the performed activities. Inspired by the idea of activity-based funding from financing the health care system^9–11^, we proposed a performance-based reimbursement model (PBRM) for collaborative clinical studies and applied it to NAPKON. We hypothesized that this approach would allow fair reimbursement irrespective of disease course, centre recruitment biases, and type of health care sector involved. To the best of our knowledge, we developed the first data-driven model for precise reimbursement of prospective multi-centre cohort studies. This might be of interest for other collaborative research networks and may also apply to randomised (pharmaceutical) clinical trials.

## Methods

### Characteristics of involved NAPKON cohorts and centres

NAPKON was initiated at the onset of the COVID-19 pandemic by the German Network of University Medicine (NUM) funded by the Federal Ministry of Education and Research (Bundesministerium für Bildung und Forschung, BMBF) to connect the German Academic Medical Centres in health research and to conduct prospective COVID-19 cohorts. NAPKON united national research activities on COVID-19 cohorts and improved the pandemic preparedness in Germany. The NAPKON cohorts have previously been described in detail^8^. In brief, it consists of three cohorts, also called “platforms”: a high-resolution, deep-phenotyping cohort of severely ill patients with COVID-19 (HAP), a population-based platform approaching former patients identified by regional health authorities (POP), and a cross-sectoral platform with acute COVID-19 patients of all stages of disease severity (SUEP). The SUEP cohort involves distinct visit schemes, distinguishing between academic medical centres (AMC; “university hospitals”) and non-academic medical centres (non-AMC; private practices, secondary and tertiary care centres). In HAP and SUEP, where acutely infected participants were recruited, study visits took place until discharge or stabilization before entering a standardized follow-up (FU) schedule. Thus, the total number of study visits depended on disease severity and length of hospital stay. Participants recruited in the POP cohort had only one on-site visit after the acute infection. Five AMC participated in more than one of the cohorts. The first enrolment for HAP, POP, and SUEP AMC was in November 2020, for the SUEP non-AMC in February 2021. This analysis included individuals recruited up to the end of the first study period (31 December 2021). The cohort studies were approved by the local ethics committee (details see “Ethics approval and consent to participate”) and were conducted in accordance with the Declaration of Helsinki. Each patient gave informed consent to participate in the study.

The cohorts were supported by infrastructure core units^8^ and the COVID-19 Exchange Data platform^12^ to increase the harmonization and methodological quality. The PBRM was primarily developed by the Interaction Core Unit (ICU), supported by the Epidemiology Core Unit (ECU), the Biosampling Core Unit (BCU), and the DICOM (imaging data) management system (DIMA).

To ensure pseudonymity, we attributed letters to the centres (A to Z, consistently used within each cohort across the results) and focused on the mean or relative reimbursement per case and centre.

### Case definition

A NAPKON participant (case) was defined as recruited if an informed consent was registered. We defined a case as reimbursable only if all relevant pages of the electronic case report form (eCRF) of the baseline and discharge visit (or the initial interview and on-site visit for the POP) were submitted by the responsible study centre. All items/activities to be reimbursed of each participant were derived from the eCRF as well as from all imaging and biosampling uploads and were provided from the data management systems. The Independent Trusted Third Party (TTP) secured and facilitated the data linkage of one reimbursement case from the data management systems without revealing individual identities or necessitating information exchange between the systems.

### Calculation of reimbursement and funding concept

The PBRM was based on a detailed Standard Operating Procedure (SOP) manual detailing each step of the process and assigning clear responsibilities for all tasks. For that purpose, we created a set of automated tools to measure quality and completeness of performed and submitted study procedures per participant and visit according to requirements defined in the study protocol. Data processing, plausibility and completeness checks were performed using Python (version 3.8.6, Python Software Foundation, Wilmington, United States) with Django (version 3.0.10, Django Software Foundation, Lawrence, United States), and R (version 4.0.2, GNU public license, provided by R Foundation, Vienna, Austria). Details of programming languages, platforms and libraries are listed in Table S1.

The reimbursement for each item originated from a comprehensive catalogue that was consensually developed beforehand by consortium members, trial specialists, and domain experts. Costs for consumables and expected time efforts of medical professionals for specific study activities and interventions (e.g., “spirometry”, “document clinical status”) were estimated and summarized for the respective activity. Time efforts were then linked to current tariff agreements in public service and added to the material expenses and applicable taxes and overheads. As a result, a lump sum for each specific study activity (termed “reimbursement item”) was reconciled among the stakeholders (centres, study coordination, coordination office of the NUM, project-executing agency). Table 1 presents exemplary compositions for reimbursement items. Each cohort then defined each type of study visits based on the reimbursement items, allowing calculations of the overall cost for one case following the activities required in the study protocol, serving as basis for the funding calculation. These considerations were also used to calculate an expected mean of reimbursement per case and cohort platform, e.g., adjusted for expected lost to FU and disease severity (here referred to as “case fee”: HAP 12,871.70€, POP 3400.76€, SUEP AMC 2904.67€, SUEP non-AMC 1071.23€).

**Table 1:** Concept of cost calculation by individual study tasks in NAPKON depicted by exemplary reimbursement items. *EDTA* ethylenediaminetetraacetic acid, *ICU* intensive care unit, medical specialist (*MS*) including specialist and senior physician, *PBMC* peripheral blood mononuclear cell, *PH* Physician, *SP* study personal.

Reimbursement item	Data-points	Time SP [min]	Salary SP [%]	Costs SP [€]	Time PH [min]	Salary PH [%]	Costs PH [€]	Time MS [min]	Salary MS [%]	Costs MS [€]	Material costs [€]	Costs overall [€]
Documentation screening and baseline	400	183	0.18	101.00	18.3	0.02	15.00					115.00
Documentation hospital ward	100	105	0.10	58.00	10.5	0.01	8.00					66.00
Documentation ICU	200	195	0.19	107.00	19.5	0.02	16.00					123.00
Document clinical status		10	0.01	5.50								5.50
Electrocardiography		10	0.01	5.50				10	0.01	11.17	1.00	17.67
PBMCs from 9 ml EDTA		120	0.12	65.97							5.00	70.97
Spirometry with plethysmograph and diffusion measurement		90	0.09	49.48	60.0	0.06	48.16	30	0.03	36.16	70.00	203.80
Transthoracic echocardiogram		15	0.01	8.25				45	0.04	50.28	25.00	83.52

The costs for individual study tasks in NAPKON are based on the assumed time effort and salary according to the tariff of study personal, physicians and medical specialists in Germany (2020). In addition, the expected material costs are considered.

For the PBRM, data items from the eCRFs were mapped to reimbursement items of the cost catalogue to represent study activities and minimum reimbursement requirements. These mapping tables were proof-read by two independent reviewers, who evaluated the proposed mappings based on epidemiological considerations. In addition, only data items passing automated plausibility and completeness checks of the respective data platforms, as well as local case reviews by the study centres were eligible for reimbursement. For biosampling or imaging meta data, distinct rules were implemented, governing reimbursement fees based on criteria like the presence and type of biosamples, number of registered aliquots or the upload of imaging series.

**Figure 1:**
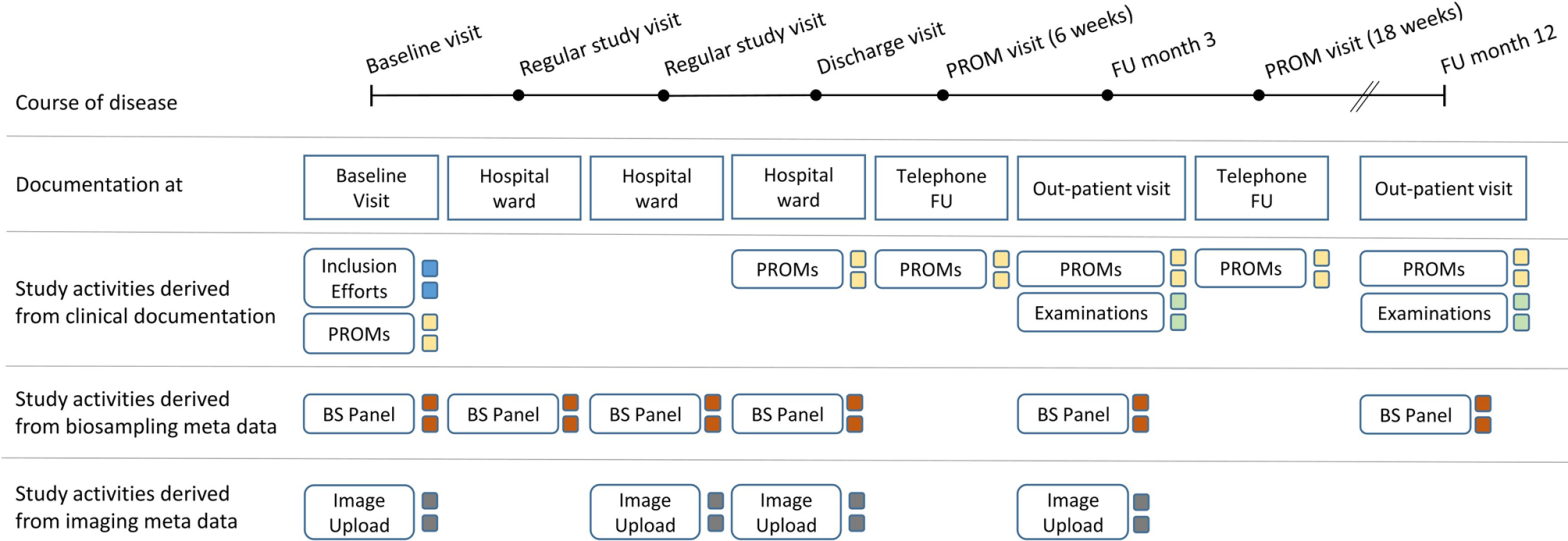
Exemplary reimbursement items across a fictional participant’s course of disease in the cross-sectoral platform (SUEP-AMC). The course of the disease and the respective visit types in the acute phase is represented by the timeline from left to right. For each visit type, different activities or documentations are designated by the study protocol. Single activities are displayed by coloured boxes aggregated by data management system. Each item is reimbursed per visit and all costs are added up to build the total reimbursement for a single case. Biosampling (BS); follow-up (FU); patient reported outcome measure (PROM).

The overall reimbursement for each case depended on various factors, including documentation, biosampling, and the upload of imaging data, and resulted as the sum of all reimbursement items (Fig. 1).

**Figure 2:**
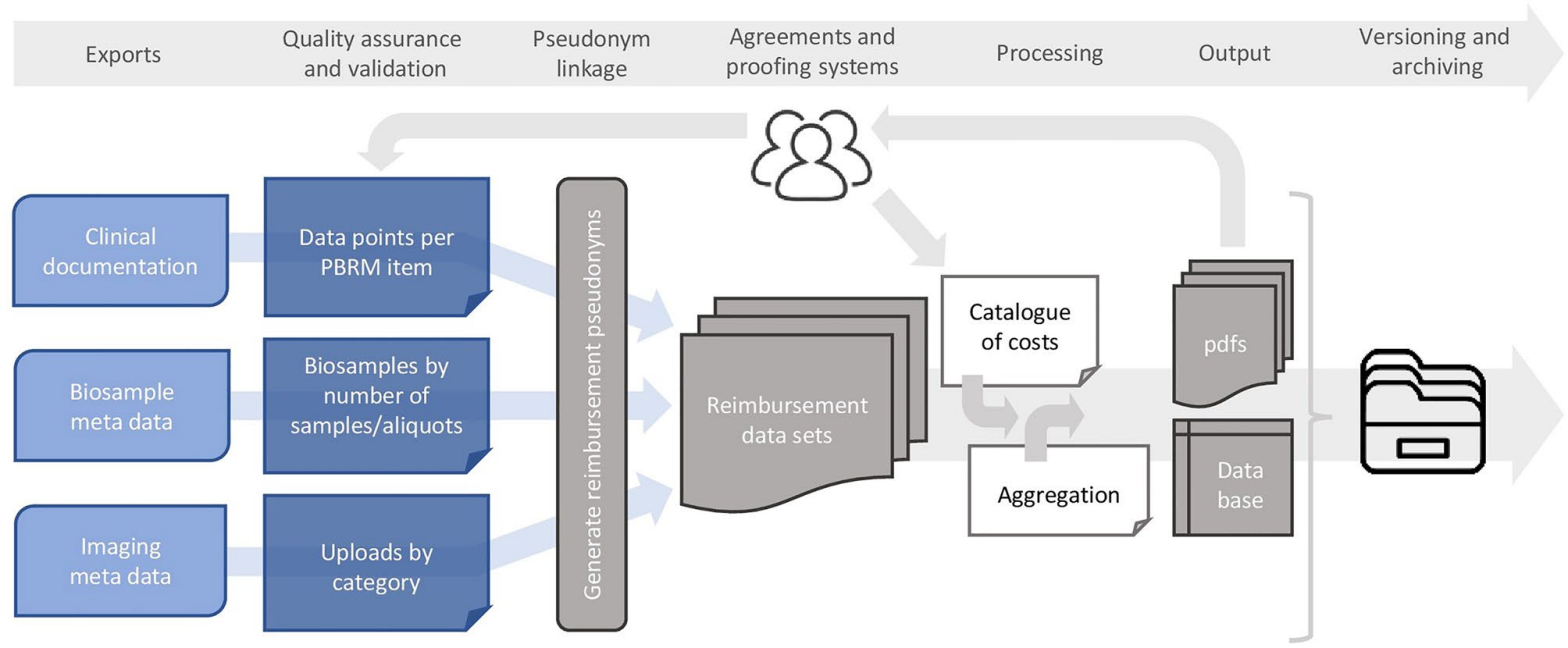
Processing steps of the tool to create the reimbursement documents using a performance-based reimbursement model. The exports of the three data management systems are checked by quality assurance and validation algorithms. The resulting data sets are joined and replenished with a catalogue of costs and information for centre aggregation to build the content of the database and the documents for the study centres. All elements of the process are versioned and archived. Performance-based reimbursement model (PBRM); portable document format (pdf).

Comprehensive reports were automatically generated, detailing results of quality checks and total reimbursement on case, centre, and cohort level, with different confidentiality and access authorization for all stakeholders (examples see Figs. S4 and S5). Announced study visits expected for the current reimbursement period that took place after documentation freeze were reimbursed based on imputation of future activities using experience values derived from the PBRM tool. Centres followed defined timelines to verify the accuracy of reports, and multiple correction loops were permitted to facilitate data improvement or rectify errors. Ultimately, data managers from all cohorts confirmed the accuracy of the reports by item-by-item checks for 10 randomly selected cases. The overall PBRM process is depicted in Fig. 2.

### Comparison to other reimbursement models

In addition to the evaluation of the PBRM output, we compared the results to both alternative funding models. For the FRM, we assumed a fixed lump sum for each actually reimbursed case, analogous to the described “case fee” per cohort. For the UFAM, personnel expenses for physicians and study nurses (rounded to the nearest quarter of a full time equivalent job) as well as consumables per case were multiplied by the targeted number of cases by each centre within the cohort (share of total targeted number of cases per cohort and centre: HAP 73.5, POP 330 or 660, SUEP AMC 98.1), thus dividing the complete funding of the cohort proportionally among all participating centres. Assuming that under the UFAM centres would have stopped the recruitment after case number completion, the model costs for comparisons were capped at the target number for over-recruiting centres. Percentages refer to the PBRM, which is used as 100% reference. SUEP non-AMC centres were not included into this analysis due to other financing structures.

### Statistical analysis

We used data generated during the PBRM implementation and performed descriptive analyses of reimbursement composition aggregated by centre and cohort. Means (M) are presented with 95%-confidence interval (95% CI) or ranges. A one-factor variance analysis (ANOVA) assessed group differences of the reimbursement models. For the statistical analysis, we used R (version 4.0.2, GNU public license, provided by R Foundation, Vienna, Austria).

### Ethics approval and consent to participate

The project was approved by the NAPKON Use & Access Committee under the application identifier: 2022-02-21_Appel_Abrechnungspipeline. All NAPKON study participants gave written informed consent. SUEP received primary approval from the ethics committee (EC) at Goethe University Frankfurt (No. 20–924), HAP from the EC of the Charité – Universitätsmedizin Berlin (No. EA2/066/20 and EA2/226/21), and POP from local ECs at study sites (Kiel, No. D 537/20, also approving the Berlin site, and Würzburg, No. 236/20). All further study sites received their local ethics vote at the respective ethics commissions.

## Results

### Study population

Illustrated in Fig. S1, 4631 participants were documented (HAP: 527 [11.4%]; POP: 2312 [49.9%]; SUEP: 1792 [38.7%]). While all cases in the HAP and POP fulfilled the minimum criteria for eCRF-derived reimbursement, 97 cases in the SUEP missed the required case review for at least one sub-form. Of the remaining 4535 cases, 19 imaging records (0.6%) and 353 biosampling records (8.1%) did not meet the criteria.

### Comparison of the PBRM between cohorts and study centres

A comprehensive overview of centre performance characteristics is provided in Table S2. The mean 3 months FU fulfilment of the HAP was 50.1% (95% CI 19.0–81.1), of the SUEP AMC 63.3% (95% CI 38.4–88.0), and of the SUEP non-AMC 69.6% (95% CI 38.3–100.0).

**Figure 3:**
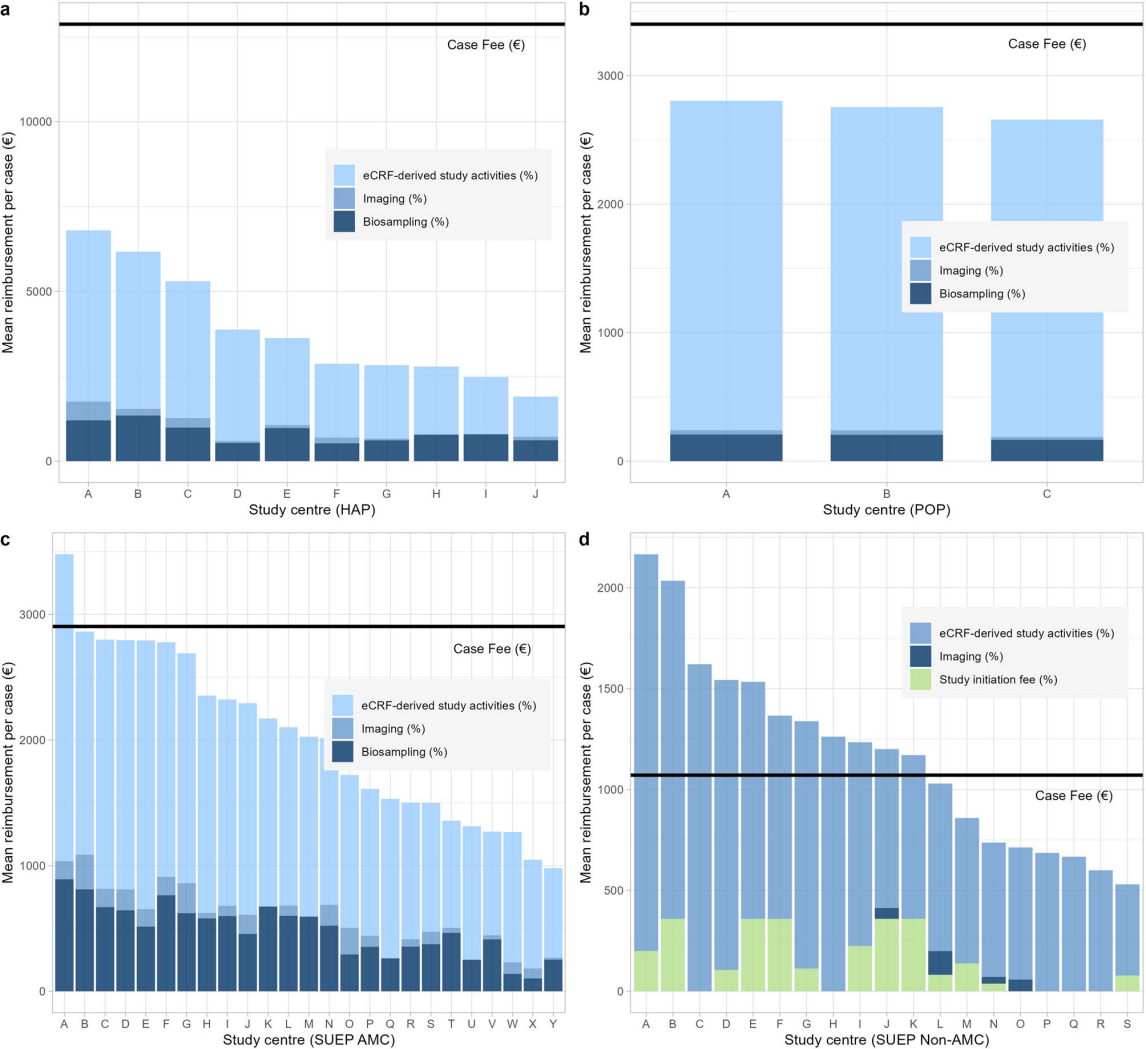
Mean reimbursement per case and study centre. (**a**) HAP centres, (**b**) POP centres, (**c**) SUEP AMC centres, (**d**) SUEP non-AMC centres. The relative proportion of the data management systems represents the contribution to the total reimbursement. The study initiation fee was granted for SUEP non-AMC centres if at least 5 cases met the reimbursement criteria. One centre of the SUEP non-AMC cohort was censored due to drop out and lack of reimbursable cases. Electronic case report form (eCRF); high-resolution platform (HAP); population-based platform (POP); cross-sectoral platform academic medical centres (SUEP AMC); cross-sectoral platform non-academic medical centres (SUEP non-AMC).

The mean case reimbursement was 3863.43€ (95% CI 1468.89–6257.96) in the HAP, 2739.71€ (95% CI 0–5839.92) in the POP, 2023.82€ (95% CI 1230.50–2817.14) in the SUEP AMC, and 1173.21€ (95% CI 645.68–1700.73) in the SUEP non-AMC. The mean reimbursement per case and centre divided by data management system is demonstrated in Fig. 3. Among the HAP centres (Fig. 3a), the contributions to the overall reimbursement ranged from 62.1% (centre J) to 84.7% (centre D) for the clinical documentation, from 0.0% (centre H) to 8.1% (centre A) for imaging and from 13.9% (centre D) to 32.5% (centre J) for biosampling. The maximum amount of the originally expected case fee achieved was 52.8% (centre A). The POP reimbursement (Fig. 3b) was more homogeneous: Clinical data caused 91.3% (centre B) to 92.9% (centre C) of the reimbursement, imaging data contributed 0.8% (centre C) to 1.3% (centre B) and biosampling contributed 6.2% (centre C) to 7.4% (centre B). All centres marginally remained below the calculated mean case fee. For the SUEP AMC centres (Fig. 3c), the contribution to the overall reimbursement ranged from 62.0% (centre B) to 82.9% (centre Q) for clinical documentation, from 0.0% (centres K, M, Q, U) to 12.3% (centre O) for imaging and from 9.8% (centre X) to 34.3% (centre T) for biosampling. One centre exceeded the expected case fee (centre A: 119.8% of the case fee), whereas all other centres remained below the expected costs. As most SUEP non-AMC centres (Fig. 3d) exclusively participated in the clinical documentation, the clinical data documentation contributed 65.6% (centre J) to 100.0% (centres C, H, P, Q, R). Four centres uploaded imaging data. Biosampling was not intended for SUEP non-AMC centres. The assumed case fee was exceeded by 57.9% of the centres (maximum: centre A with 202.2% of the case fee).

As the nature of the PBRM suggests, the reimbursement in the SUEP and HAP cohorts increased with the FU compliance (Fig. S2). Detailed information on the contribution of (FU) visit types in the course of disease to total eCRF-derived reimbursement is provided in Fig. S3.

### Comparison of reimbursement models

**Figure 4:**
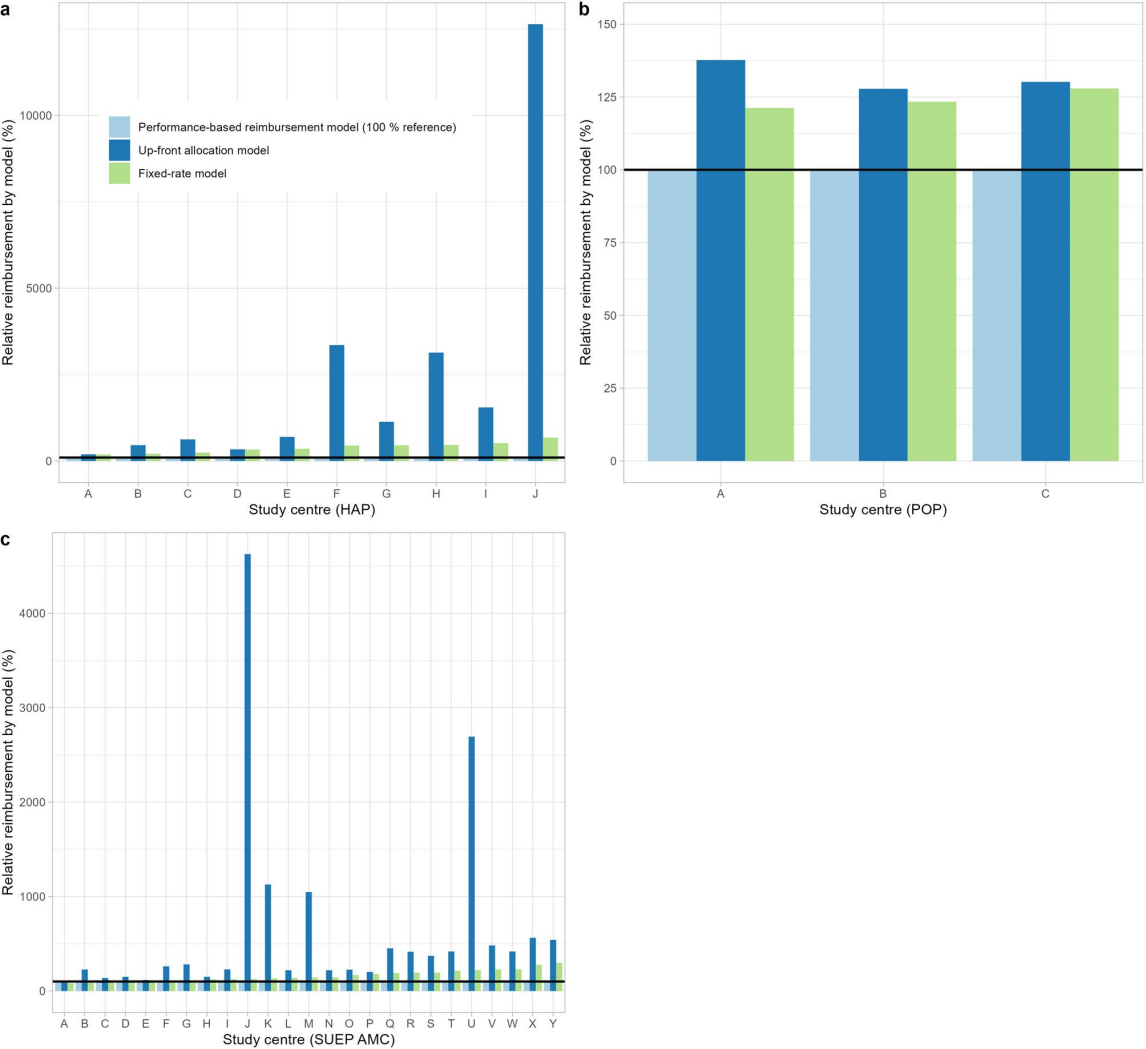
Comparing reimbursement models relative to PBRM. (**a**) HAP centres, (**b**) POP centres, (**c**) SUEP AMC centres. The comparison includes our novel performance-based reimbursement model (PBRM) which is chosen to be the reference for each individual centre (100%) and the theoretical application of reimbursement using the UFAM and FRM. High-resolution platform (HAP); fixed-rate model (FRM); population-based platform (POP); cross-sectoral platform academic medical centres (SUEP AMC); up-front allocation model (UFAM).

Figure 4 shows the comparison of our PBRM with the alternative approaches (UFAM and FRM), with PBRM as reference. Within the HAP cohort, centre A (164 cases, case mean of 6797.53€) and centre D (102 cases, case mean of 3876.32€) significantly exceeded the planned number of 73.5 cases. In contrast, centre J was only reimbursed for four cases (case mean 1901.91€). With all centres undercutting the expected case fee, the FRM would have overfunded the centres from 189.4% (centre A) to 676.8% (centre J), compared to the 100% PBRM reference. With centres A and D being capped at the target number of cases, the UFAM would have provided 192.4% and 337.4% of the PBRM, respectively. Centres that have not met their recruitment target (specifically, centres F, H, and J) would have received 3352.7%, 3135.5%, and 12,635.8% of the PBRM funding, respectively, with centre J (4 cases) receiving a mean reimbursement per case of 240,320.49€. Calculating a mean number of cases that could be recruited with an assumed budget of 10,000€, the PBRM enabled 3.0 (95% CI 2.3–3.8) cases per unit, the FRM 0.8 (95% CI 0.8–0.8) cases per unit and the UFAM 0.3 (95% CI 0.2–0.5) cases per unit (ANOVA: p < 0.001).

The POP centres A, B, and C were reimbursed for 301, 348 and 1663 cases yielding a mean reimbursement of 2804.74€, 2756.18€ and 2658.20€ per case, respectively. As the PBRM result of all centres was lower as compared to the expected case fee and with centres B and C being capped at the respective target number of cases, application of the FRM (121.3%, 123.4% and 127.9% of the PBRM, respectively) and the UFAM (137.7%, 127.8%, and 130.2% of the PBRM, respectively) would have been less cost efficient. Considering a mean number of cases, a budget of 10,000€ would have enabled 3.7 (95% CI 3.5–3.8) cases according to the PBRM, 2.9 (95% CI 2.9–2.9) cases according to the FRM and 2.8 (95% CI 2.6–3.0) according to the UFAM (ANOVA: p < 0.001).

Among the high-performing SUEP AMC centres, centres A and E recruited 86 and 110 cases and reached a mean case reimbursement of 3479.29€ and 2793.60€, respectively. For centre Y, 60 cases were reimbursed with 980.30€ per case, whereas centre J was reimbursed for 3 cases with a mean reimbursement of 2293.73€. Due to the overrun of the estimated average costs, centre A would have received 83.5% under the FRM as compared to the PBRM. In contrast, centres B to Y (below the case fee) would have received 101.4% to 296.3% more funding according to the FRM. The use of the UFAM would have resulted in overfunding all centres, up from 106.4% (centre A) of the PBRM. Low recruiting outlier centres such as centre J (3 cases) and U (9 cases) would have received the same funding as high recruiters, i.e. 4626.0% and 2692.99% of the PBRM. The mean number of cases per 10,000 € for the SUEP AMC centres is 5.6 (95% CI 4.8–6.3) cases for the PBRM, 3.4 (95% CI 3.4–3.4) cases for the FRM and 1.8 (95% CI 1.5–2.1) cases for the UFAM (ANOVA: p < 0.001).

## Discussion

While a performance-based approach is common in financing health care activities^9,13^, there are only few studies discussing funding allocation models as a management tool in collaborative clinical research^1,14,15^. Under specific circumstances, e.g., studies on a well-characterized disease, long-term collaborations known for their reliability and rather small consortia, it may be appropriate and preferable to consider using the FRM or UFAM for the funding allocation, especially due to a slim administrative procedure. However, in the unknown settings of a new pandemic and potential variability of centre performance, we proposed a novel data-driven performance-based reimbursement model serving these needs in a COVID-19 multi-centre cohort study. Our hypothesis was that this model would enable fair, transparent, and adequate allocation of funding to the NAPKON centres.

Upon implementing the PBRM to NAPKON, we observed a considerable heterogeneity in case reimbursement and underachievement of expected case fees among HAP and SUEP centres. This discrepancy may be attributed to several factors: the specific recruitment engagement and clinical protocol (adherence), relatively low participant engagement regarding follow-up compliance, the diverse nature of the participating research centres, and a lower-than-anticipated case severity. New virus variants of concern, enhanced therapeutic interventions and changes in the population’s immunity presented with different disease burden of COVID-19^7,16,17^, thus resulting in a deviation of length of hospital stay and variety of reimbursement-relevant items. In contrast, the three centres in the POP cohort had a relatively similar mean reimbursement per case closer to the case fee. This might be because the study setting is quite different. While in the HAP and SUEP cohort participants were enrolled during their hospital stay, POP participants were invited by collaborating heath care authorities and visited the study centres primarily for study procedures, contributing to a more homogeneous performance. For HAP and SUEP, we conclude that the PBRM output can serve as a surrogate to compare case severity, execution quality, and overall effort between study centres. In this sense, PBRM might also serve as a performance indicator measuring the overall quality of a study centre and may thus be helpful in successfully coordinating complex multi-centre studies^14,18,19^.

Comparing the three reimbursement models (FRM, UFAM, and PBRM), the model-dependent funding allocation differed strongly. Regarding recruitment and performance outliers, centres with few recruited participants, (e.g., centre J in SUEP and HAP) would have received the same UFAM funding as high-performing centres exceeding the target number of cases (e.g., HAP centre A, SUEP AMC centres A, E, P). Without additional financial incentives, the high-performing centres might have stopped their recruiting engagement resulting in delayed study completion and—in the worst case—in the inability to achieve the required case number. This situation could weaken the statistical power necessary to answer research questions—at least in case of UFAM. Therefore, using UFAM would have led to equal treatment of unequal performance, raising major concerns in terms of fairness and competition incentives. The FRM would be closer in addressing recruitment disparities and would have simplified the administration compared to the UFAM. Nevertheless, it would not have considered relevant variations within reimbursement cases. Especially for the HAP and SUEP cohorts, the PBRM shifts the funding process towards a more precise and cost-efficient allocation of public research funds.

In previous studies, the detailed description of a performance-based allocation of funding is scarce. Specifically, Gist and Langley implemented a project management tool in a multi-national clinical trial in order to “link expenditure to activities at geographically separate trial sites”. They defined diagnostics, participant reimbursement and enrolment as target activities and connected these activities to the used budget^15^. Furthermore, the monitoring of data quality in terms of missing information is emphasized by Nasser et al. suggesting a need for an efficient and strict control mechanism and minimum quality check before accepting the documentation of a participating centre^20^. Despite the implementation of a mutual and iterative learning process among all stakeholders within NAPKON, monitoring the budget spent and the achieved target activities improved transparency and confidence among the partners. Therefore, the PBRM served as a management tool in NAPKON and enabled the efficient resource allocation among all centres.

However, there are limitations of the implementation of the PBRM. Firstly, the approach requires a substantial restructuring of the funding system and the way of thinking in (university) clinical research. To prepare a study, initial financial support for the set-up and in-time reimbursement of performed activities is required (and demanded) by the participating centres. Therefore, start-up fees need to be balanced with PBRM, while avoiding double reimbursement by different funding layers. To achieve timely reimbursement, further automation of the PBRM data pipeline, calculation tool and quality assurance process are necessary. This also requires that anticipated reimbursements serve as a bail to prolong contracts with research staff, as the compensation for their work is delivered with a time-delay. Furthermore, the automation aims to reduce personnel and time resources needed for processing steps. Secondly, the notable benefits of PBRM – cost-efficiency and transparency – also present a limitation: the overall funding received by participating centres may be lower than expected based on previous experience if quality or quantity of provided data and biosamples fall below expectations. If e.g. several attempts to execute distinct study activities that are relevant for reimbursement fail – either to participant-related or technical reasons – these unsuccessful but time-consuming measures are not compensated for and therefore pose a significant financial risk to the study centres. In addition, some activities are not displayed in the funding (e.g., efforts like reaching participants for FU schedule appointments). In the PBRM, the respective study centres carry more operative risks, like unexpected personnel outage, administrative/regulatory delays, force majeure. For future studies, we aim to enhance the cost catalogue – e.g., the reimbursement per item – to better include safety margins and consider activities indirectly connected with a trial. Thirdly, within NAPKON, complexity was added by the necessity to book all fees within the same fiscal year: e.g., PBRM calculation, quality assurance, feedback loops, accounting, submission of invoices need considerable preparation time, resulting in an unobserved time window between the date of PBRM calculation and the end of the fiscal year. We therefore needed to extrapolate the future reimbursement to rationally justify our assumptions. Finally, while it is possible that the higher initial funding in the UFAM would have allowed allocating more resources to the project, it must be considered that all university centres received substantial funding and it is doubtful that further increasing up-front funding would have influenced recruitment by the orders of magnitude required to come anywhere near the cost-efficiency of the PBRM.

## Conclusion

In our analysis, we observed the following major advantages of the PBRM:

(i) **Flexibility**: Our concept can be applied to multi-centre cohorts with distinct study protocols and diverse populations, even for unknown diseases, as a homogeneous and robust reimbursement framework.
(ii) **Efficient allocation of resources**: Funding is defined by quality and quantity of the respective centre performance. This makes the PBRM transparent, fair, and easily verifiable.
(iii) **Quality assurance and control function**: The model enables the study coordination to combine quality checks with reimbursement rules and therefore emphasize certain procedures in the study protocol. It supports monitoring the performances of individual study centres and a budgetary control mechanism for the project coordinators. Additionally, it implies incentives for a qualitative and comprehensive documentation for the centres themselves.

While our results comparing PBRM to other funding models are based on projections and necessitate validation in a large-scale prospective cluster-randomized trial, this endeavour would require a very large number of participating centres and is unlikely to be feasible in the near future. Our novel concept may serve as a template for a future transition to a more precise reimbursement within the clinical study landscape. Especially in heterogeneous study settings, among newly composed research collaborations and in clinical studies that are performed within the conventional structures of healthcare delivery, PBRM might have significant advantages compared to other funding methods, while in small studies or established long-term collaborations the benefits of the slim administrative procedures of UFAM and FRM might prevail. In the future, we aim to further develop the tool by focusing on automation, generalization, real-time updates, and reporting options. The proposed PBRM concept and the cost catalogue can be adapted to be used in other multi-centre, multi-study, and (multi-)national settings.

### Supplementary Information

Supplementary Information.

## Data Availability

The datasets generated and analysed during the current study are not publicly available due to confidentiality reasons but are available from the corresponding author upon reasonable request.
