# Supplementary material for: A precise performance-based reimbursement model for the multi-centre NAPKON cohorts – development and evaluation
Source: Sci Rep. 2024 Jun 13;14:13607. doi: 10.1038/s41598-024-63945-5 (PMC11176345; doi:10.1038/s41598-024-63945-5)
Supplement: Supplementary file 1 — Supplementary Information. [file 41598_2024_63945_MOESM1_ESM.pdf]

Supplementary material to

A precise performance-based reimbursement model for the multi-centre NAPKON cohorts – development and evaluation

Katharina S. Appel[1,2], Chin Huang Lee[2], Susana M. Nunes de Miranda[2], Daniel Maier[1,3], Jens-Peter Reese[4,5], Gabriele Anton[6], Thomas Bahmer[7,8], Sabrina Ballhausen[7], Beate Balzuweit[9], Carla Bellinghausen[10], Arne Blumentritt[11], Markus Brechtel[2], Irina Chaplinskaya-Sobol[12], Johanna Erber[13], Karin Fiedler[1,2], Ramsia Geisler[1,2], Ralf Heyder[14], Thomas Illig[15], Mirjam Kohls[4], Jenny Kollek[16], Lilian Krist[17], Roberto Lorbeer[18,19,20], Olga Miljukov[4,5], Lazar Mitrov[21], Carolin Nürnberger[4,5], Christian Pape[12], Christina Pley[22], Christian Schäfer[23], Jens Schaller[18,19], Mario Schattschneider[23], Margarete Scherer[1], Nick Schulze[1,2], Dana Stahl[11], Hans Christian Stubbe[24,25], Thalea Tamminga[7], Johannes Josef Tebbe[26,27], Maria J.G.T. Vehreschild[28], Silke Wiedmann[14], Jörg Janne Vehreschild[1,2,29] on behalf of the NAPKON Study Group

1 Goethe University Frankfurt, University Hospital, Center for Internal Medicine, Medical Department 2 (Hematology/Oncology and Infectious Diseases), Frankfurt, Germany
2 University of Cologne, Faculty of Medicine and University Hospital of Cologne, Department I for Internal Medicine, Cologne, Germany
3 German Cancer Consortium (DKTK), Partner Site Frankfurt/Mainz and German Cancer Research Center (DKFZ), Heidelberg, Germany
4 University of Würzburg, Institute for Clinical Epidemiology and Biometry, Würzburg, Germany
5 University Hospital Würzburg, Institute for medical Data Science, Würzburg, Germany
6 Medical School OWL,Bielefeld University, Bielefeld, Germany
7 Internal Medicine Department I, Pneumology Section, University Hospital Schleswig-Holstein Campus Kiel, Kiel, Germany
8 German Center for Lung Research (DZL), Airway Research Center North (ARCN), Grosshansdorf, Germany
9 Charité – Universitätsmedizin Berlin, Corporate Member of Freie Universität Berlin and Humboldt-Universität zu Berlin, Department of Infectious Diseases, Respiratory Medicine and Critical Care, Berlin, Germany
10 Department I of Internal Medicine, Goethe University Frankfurt, University Hospital Frankfurt, Frankfurt am Main, Germany
11 Independent Trusted Third Party of the University Medicine Greifswald, Ellernholzstraße 1-2, 17475 Greifswald, Germany
12 Department of Medical Informatics at the University Medical Center Göttingen, Göttingen, Germany
13 TUM School of Medicine and Health, Department of Clinical Medicine – Clinical Department for Internal Medicine II, Technical University of Munich, University Medical Center, Munich, Germany
14 Charité – Universitätsmedizin Berlin, corporate member of Freie Universität Berlin and Humboldt-Universität zu Berlin, NUM Coordination Office, Charitéplatz 1, 10117 Berlin, Germany
15 Hannover Medical School, Hannover Unified Biobank, Hannover, Germany
16 Charité - Universitätsmedizin Berlin, Corporate Member of Freie Universität Berlin and Humboldt-Universität zu Berlin, Department of Infectious Diseases, Respiratory Medicine and Critical Care, Clinical Trial Unit Berlin, Berlin, Germany
17 Institute of Social Medicine, Epidemiology and Health Economics, Charité – Universitätsmedizin Berlin, corporate member of Freie Universität Berlin and Humboldt-Universität zu Berlin, Berlin, Germany
18 Deutsches Herzzentrum der Charité, Institute of Computer-assisted Cardiovascular Medicine, Berlin, Germany
19 Charité – Universitätsmedizin Berlin, corporate member of Freie Universität Berlin and Humboldt Universität zu Berlin, Berlin, Germany
20 Department of Radiology, Ludwig-Maximilians-University Hospital, Munich, Germany
21 University of Cologne, Faculty of Medicine and University Hospital of Cologne, Department I of Internal Medicine, Center for Integrated Oncology Aachen Bonn Cologne Duesseldorf, Cologne, Germany
22 Charité – Universitätsmedizin Berlin, corporate member of Freie Universität Berlin and Humboldt-Universität zu Berlin, Clinical Trial Office, Berlin, Germany
23 Institute of Clinical Chemistry and Laboratory Medicine, University Medicine Greifswald, Greifswald, Germany
24 Department of Medicine II, University Hospital, LMU Munich, Munich, Germany
25 German Center for Infection Research (DZIF), Partner-Site Munich, Munich, Germany
26 Hospital Lippe, Department of Gastroenterology and Infectious Diseases, Lippe, Germany
27 Bielefeld University, Medical School OWL, Bielefeld, Germany
28 Goethe University Frankfurt, University Hospital Frankfurt, Department II of Infectious Diseases, Frankfurt am Main, Germany
29 German Center for Infection Research (DZIF), Partner-Site Cologne-Bonn, Cologne, Germany

Corresponding authors

Jörg Janne Vehreschild
Goethe University Frankfurt, University Hospital, Center for Internal Medicine, Medical Department 2, Frankfurt, Germany
Theodor-Stern-Kai 7, 60596 Frankfurt am Main
Tel.: +49 151 17192218, Email: j.vehreschild@med.uni-frankfurt.de

Katharina S. Appel
Goethe University Frankfurt, University Hospital, Center for Internal Medicine, Medical Department 2, Frankfurt, Germany
Theodor-Stern-Kai 7, 60596 Frankfurt am Main
Tel.: +49 151 1719 0565; Email: appel@med.uni-frankfurt.de

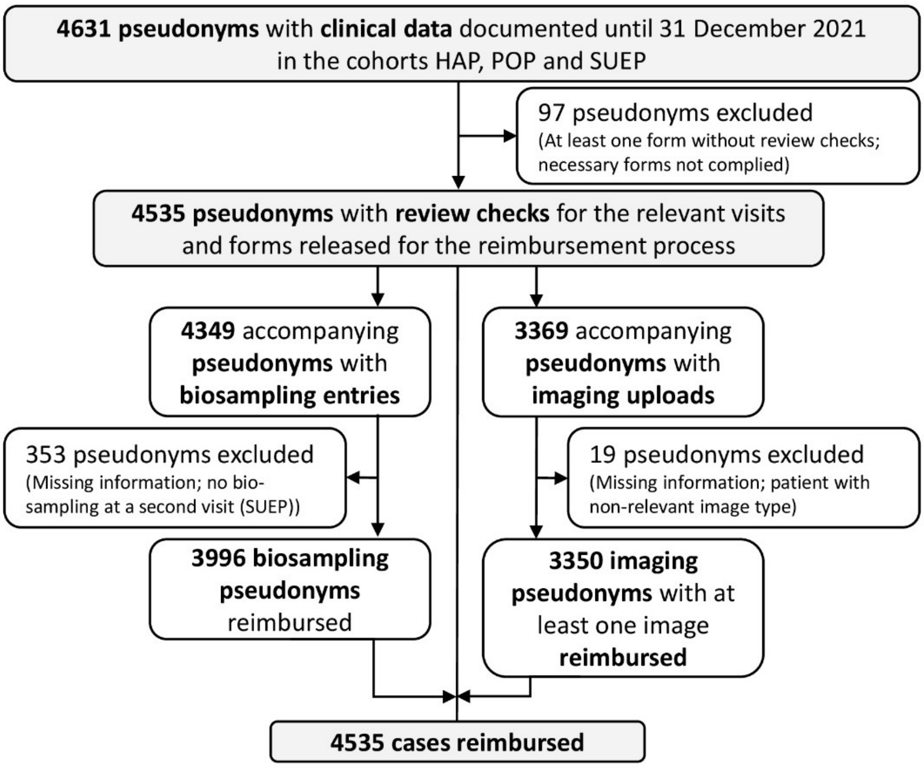

Figure S1. Flowchart of reimbursed cases and reasons for exclusion.

For each data management system, a different set of rules for quality assurance and validation is applied. Pseudonyms who did not fulfil these criteria could not be reimbursed. The reimbursement of the clinical data is the prerequisite for the accompanying reimbursement of the image uploads and biosampling for each accompanying pseudonym (different pseudonyms in each data management system). As biosampling is not feasible or images are not available (and planned) for some participating (non-university) study sites, there are less pseudonyms in the imaging or biosampling management system than in the clinical data management system, especially in the SUEP cohort. High-Resolution Platform (HAP); Population-Based Platform (POP); Cross-Sectoral Platform (SUEP).

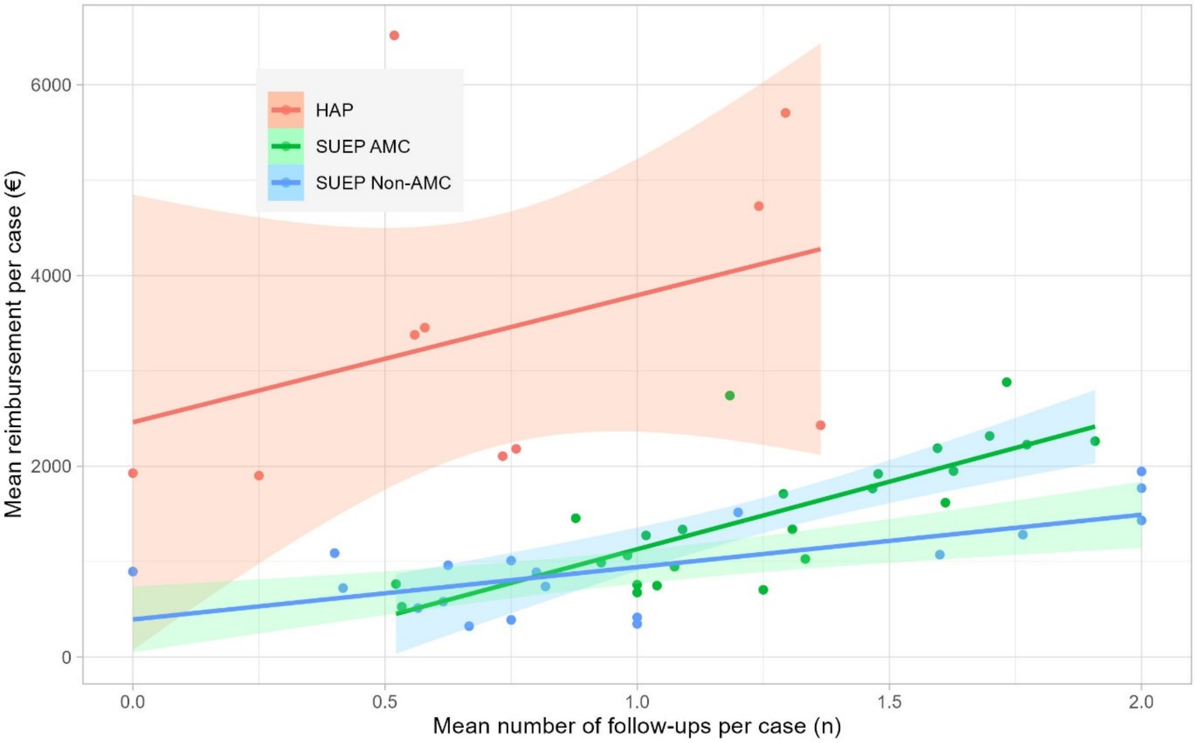

Figure S2. Mean reimbursement per case increases with the number of follow-ups.

The relationship between the average reimbursement per case and mean number of follow ups is displayed using a regression line and standard error by platform. We observed a positive correlation (Peason's correlation coefficient) for clinical data reimbursement with compliance to the FU for the SUEP (SUEP AMC: r=0.77, p<0.001; SUEP non-AMC: r=0.69, p=0.001). The variance in the HAP FUs was high (r=0.36, p=0.308). The Population-Based Platform did not have a follow-up in the first funding period. High-Resolution Platform (HAP); Cross-Sectoral Platform academic medical centres (SUEP AMC); Cross-Sectoral Platform non-academic medical centres (SUEP AMC).

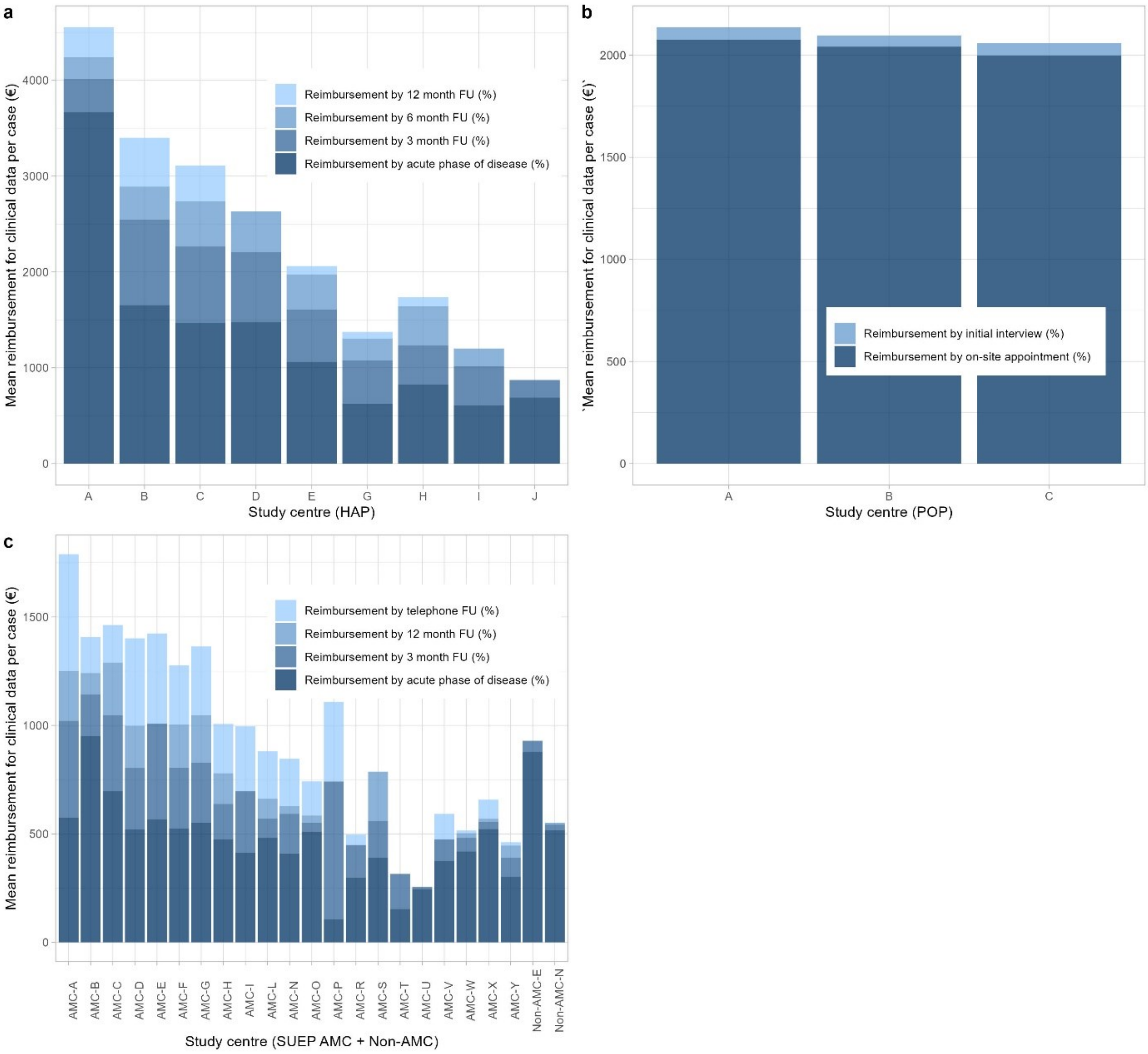

Figure S3. Contribution of visit types in the course of disease to total clinical documentation-based reimbursement of each study centre.

Each colour shaded section reflects the types of visits applicable by the respective study protocol of the cohort. Section **(a)** and **(b)** show the composition of HAP and POP, SUEP non-AMC are embedded together with SUEP AMC in **(c)**, as there are few centres with finished cases. Due to the longitudinal design, the follow-up visits of certain cases might not have been applicable. This depiction only includes cases who had the chance to participate in all follow-up visits. Follow-up (FU); High-Resolution Platform (HAP); Population-Based Platform (POP); Cross-Sectoral Platform academic medical centre (SUEP AMC); Cross-Sectoral Platform non-academic medical centre (SUEP Non-AMC).

NAPKON
NATIONALES
PANDEMIE
KOHORTEN
NETZ

Charite – Universitätsmedizin Berlin
Koordinierungsstelle Netzwerk Universitätsmedizin
Chariteplatz 1
10117 Berlin

Betreff: 01KX2021; NUM Case Fees; NAPKON; IA 88116131 (NAPKON)

Einzelpatient:innennachweis für Zentrum [redacted]

Dokument-Nummer: 2204051-0206-0001 **05.04.2022**

Patient:innen ID: pheno_[redacted]

Datum		Leistungen	Einzelvergütung	Abgerechnete Anzahl	Gesamte Vergütung
**25.02.2021**	**1**	Doku Allgemein Screening + Baseline	115,29	1	115,29
	**2**	Entscheidung über Studieneinschluss	5,59	1	5,59
	**3**	Patient*inneninformation, Einwilligung	32,33	1	32,33
	**4**	Prüfung Ein- und Ausschlusskriterien	26,76	1	26,76
**01.03.2021**	**5**	Doku Allgemein Visite Normalstation	66,15	1	66,15
**05.03.2021**	**6**	Doku Allgemein Visite Normalstation	66,15	1	66,15
**12.04.2021**	**7**	Doku Allgemein Telefon-Follow-Up	20,79	1	20,79
	**8**	Mindestpauschale PROM	22,71	1	22,71
	**9**	Terminvereinbarung Patient:in	8,25	1	8,25

Gesamtkosten: 364,02 €

Major: v1.2.2
Minor: v1.0.2204052242

1 von 1

Figure S4. Exemplary reimbursement document provided to the NAPKON study centres on the single patient level.

Additionally, an aggregation by centre (Figure S5) and an aggregation by platform is provided, respectively.

NAPKON
NATIONALES
PANDEMIE
KOHORTEN
NETZ

Charite – Universitätsmedizin Berlin
Koordinierungsstelle Netzwerk Universitätsmedizin
Charitéplatz 1
10117 Berlin

Betreff: 01KX2021; NUM Case Fees; NAPKON; IA 88116131 (NAPKON)

Leistungsübersicht für [redacted]

Dokument-Nummer: 2204051-0209-0001 **05.04.2022**

Leistungsübersicht:

	Aktivität	Einzelvergütung	Abgerechnete Anzahl	Gesamte Vergütung
1	Arbeitsfähigkeit (2/6)	13,62	3	13,62
2	Arbeitsfähigkeit (3/6)	13,62	2	13,62
3	Arbeitsfähigkeit (4/6)	13,62	21	190,68
4	Arbeitsfähigkeit (5/6)	13,62	3	34,05
5	CT-Kranial	28,53	4	114,12
6	CT-Körperregion unsicher	28,53	8	228,24
7	CT-Thorax	28,53	3	85,59
8	Doku Allgemein Screening + Baseline	115,29	59	6.802,11
9	Doku Allgemein Telefon-Follow-Up	20,79	4	83,16
10	Doku Allgemein Visite Ambulant	66,15	3	198,45
11	Doku Allgemein Visite Intensivstation	122,86	40	4.914,40
12	Doku Allgemein Visite Normalstation	66,15	86	5.688,90
13	EQ-5D-5L	13,62	32	435,84
14	Entscheidung über Studieneinschluss	5,59	60	335,40
15	PHQ-4	9,08	32	290,56
16	PROMIS Global Health	22,71	32	726,72
17	PROMIS Kognitive Funktion	9,08	32	290,56

Major: v1.2.2
Minor: v1.0.2204052242

	Aktivität	Einzelvergütung	Abgerechnete Anzahl	Gesamte Vergütung
18	Patient*inneninformation, Einwilligung	32,33	60	1.939,80
19	Prüfung Ein- und Ausschlusskriterien	26,76	60	1.605,60
20	Röntgen-Thorax	28,53	60	1.711,80
21	Screeningfragen zu Schmerz, Dyspnoe, Fatigue	24,97	1	24,97
22	Screeningfragen zu Schmerz, Dyspnoe, Fatigue (10/11)	24,97	8	181,60
23	Screeningfragen zu Schmerz, Dyspnoe, Fatigue (3/11)	24,97	23	156,63
24	Terminvereinbarung Patient:in	8,25	35	288,75
25	Vitalzeichenkontrolle	5,50	2	11,00

Kumulierte Kosten: 26.366,17 €

Projektpauschalen 20%: 5.273,23 €

Kumulierte Gesamtkosten: 31.639,40 €

Entsprechnung in Standardfällen (2.904,67 €): 10.89

Gesamtkosten für 11 Standardfälle : 31.951,37 €

Weitere Informationen über zukünftig projizierte Kosten werden separat ausgewiesen.

Enthaltene Pseudonyme:

Abs. Anzahl der Fälle 60

[redacted]

Major: v1.2.2
Minor: v1.0.2204052242

Figure S5. Exemplary reimbursement documents provided to the NAPKON study centres on the centre aggregation level.

Additionally, a single patient overview and an aggregation by platform is provided to the centres and the platform coordination, respectively.

Table S1. Programming languages, platforms, and libraries.

Programs	Version	Producer	Reference
**Python**	3.8.6	Python Software Foundation	https://www.python.org/
jinja2	2.11.0	Pallets	https://jinja.palletsprojects.com/
numpy	1.19.4	NumPy	https://numpy.org/
pandas	1.3.1	NumFOCUS, Inc.	https://pandas.pydata.org/
pdfkit	1.0.0	PyPI	https://pypi.org/project/pdfkit/
**R**	4.0.2	The R Foundation	https://www.r-project.org/
data.table	1.14.0	Matt Dowle, Arun Srinivasan	https://cran.r-project.org/web/packages/data.table/index.html
dplyr	1.0.5	Hadley Wickham, Romain François, Lionel Henry, Kirill Müller, RStudio	https://dplyr.tidyverse.org/
lubridate	1.7.10	Vitalie Spinu, Garrett Grolemund, Hadley Wickham	https://lubridate.tidyverse.org/
readxl	1.3.1	Hadley Wickham, Jennifer Bryan, RStudio	https://readxl.tidyverse.org/
readr	2.0.1	Hadley Wickham, Jim Hester, Jennifer Bryan, RStudio	https://readr.tidyverse.org/
**RStudio**	1.3.1093	posit	https://posit.co/
**Django**	3.0.10	Django Software Foundation	https://www.djangoproject.com/
**Nextcloud**	21.0.9	Nextcloud GmbH	https://nextcloud.com/
**Keycloak**	18.0.0	WildFly, a division of Red Hat	https://www.keycloak.org/

Table S2: Recruitment and reimbursement characteristics by cohort and centre.

Centre	First enrolment (Month/Year)	Documented cases (n)	Reimbursed cases (n)	Average reimburse-ment per case (€)	Fulfilment rate follow up month 3 (%)	Fulfilment rate follow up month 6 (%)	Fulfilment rate follow up month 12 (%)	Fulfilment rate follow up by telephone (%)
**High-Resolution Platform**								
Centre A	11/2020	164	164	6,797.53	37.1	42.0	26.2	
Centre B	12/2020	34	34	6,170.33	88.0	77.3	33.3	
Centre C	1/2021	29	29	5,299.43	86.4	66.7	41.7	
Centre D	2/2021	102	102	3,876.32	40.9	33.9	0.0	
Centre E	2/2021	38	38	3,626.85	44.0	34.6	5.0	
Centre F	11/2021	10	10	2,867.22	0.0	0.0	0.0	
Centre G	2/2021	30	30	2,827.40	52.0	44.4	50.0	
Centre H	2/2021	11	11	2,787.08	72.7	54.5	25.0	
Centre I	4/2021	25	25	2,480.22	54.5	23.8	0.0	
Centre J	3/2021	4	4	1,901.91	25.0	0.0	0.0	
**Population-Based Platform**								
Centre A	3/2021	301	301	2,804.74				
Centre B	1/2021	348	348	2,756.18				
Centre C	11/2020	1663	1663	2,658.20				
**Cross-Sectoral Platform – academic medical centres (AMC)**								
Centre A	1/2021	88	86	3,479.29	84.9		78.6	74.9
Centre B	1/2021	50	49	2,864.11	52.9		33.3	25.7
Centre C	12/2020	84	83	2,799.78	80.3		78.1	26.4
Centre D	1/2021	80	76	2,795.10	75.7		53.8	59.4
Centre E	2/2021	114	110	2,793.60	86.0		0.0	51.7
Centre F	1/2021	45	44	2,778.53	89.5		61.5	43.4
Centre G	1/2021	42	42	2,691.39	70.6		60.0	93.1
Centre H	11/2020	91	90	2,354.12	63.0		19.5	33.4
Centre I	3/2021	61	60	2,323.38	74.1		17.4	41.4

Centre J	12/2021	3	3	2,293.73	100.0	0.0	50.0
Centre K	6/2021	19	13	2,171.67	46.2	0.0	36.4
Centre L	2/2021	69	69	2,102.35	56.5	15.0	41.4
Centre M	7/2021	15	15	2,026.79	77.8	0.0	41.5
Centre N	1/2021	73	72	2,015.81	70.8	29.0	32.2
Centre O	12/2020	82	82	1,721.52	52.1	46.2	26.4
Centre P	4/2021	112	100	1,611.16	30.5	0.0	29.7
Centre Q	5/2021	47	46	1,532.44	57.1	0.0	9.8
Centre R	3/2021	51	51	1,503.03	62.5	0.0	12.6
Centre S	1/2021	60	57	1,500.83	64.0	55.2	3.6
Centre T	2/2021	60	56	1,357.91	38.9	0.0	2.3
Centre U	3/2021	9	9	1,313.39	50.0	0.0	4.8
Centre V	2/2021	54	52	1,271.10	38.3	11.1	12.5
Centre W	2/2021	60	60	1,267.27	61.4	66.7	10.0
Centre X	12/2020	54	54	1,046.92	59.2	24.4	25.3
Centre Y	2/2021	62	60	980.30	37.5	0.0	5.1
**Cross-Sectoral Platform – non-academic medical centres (non-AMC)**							
Centre A	8/2021	9	9	2,165.96	100.0	0.0	90.9
Centre B	10/2021	5	5	2,034.76	100.0	100.0	100.0
Centre C	8/2021	2	2	1,621.10	100.0	0.0	100.0
Centre D	8/2021	18	17	1,542.94	100.0	0.0	100.0
Centre E	3/2021	6	5	1,534.00	100.0	0.0	40.0
Centre F	9/2021	6	5	1,366.25	100.0	0.0	100.0
Centre G	8/2021	17	16	1,338.55	72.7	0.0	100.0
Centre H	12/2021	1	1	1,261.97	0.0	0.0	0.0
Centre I	10/2021	14	8	1,234.53	62.5	0.0	62.5
Centre J	11/2021	5	5	1,200.46	100.0	0.0	80.0
Centre K	4/2021	5	5	1,170.39	66.7	0.0	14.3
Centre L	7/2021	22	22	1,029.63	89.5	0.0	66.7
Centre M	10/2021	14	13	858.90	33.3	0.0	23.1
Centre N	2/2021	50	48	736.69	34.9	16.7	11.3
Centre O	11/2021	1	1	712.82	100.0	0.0	0.0

Centre P	12/2021	4	4	685.66	0.0		0.0	75.0
Centre Q	12/2021	2	2	666.75	0.0		0.0	100.0
Centre R	10/2021	3	3	599.77	100.0		0.0	50.0
Centre S	8/2021	25	23	529.81	61.9		0.0	2.8

Fulfilment rates are calculated without deceased patients. If the recruitment launch of a centre was comparably late, the conduction of a certain follow-up might not (yet) have been feasible.
